# Laser sintering of gravure printed indium tin oxide films on polyethylene terephthalate for flexible electronics

**DOI:** 10.1038/s41598-018-38043-y

**Published:** 2019-02-11

**Authors:** A. A. Serkov, H. V. Snelling, S. Heusing, T. Martins Amaral

**Affiliations:** 1 University of Hull, Physics - School of Mathematics and Physical Sciences, Kingston Upon Hull, HU6 7RX UK; 2 INM – Leibniz Institute for New Materials, Department of Optical Materials, 66123 Saarbruecken, Germany

## Abstract

Tin doped indium oxide (ITO) thin films provide excellent transparency and conductivity for electrodes in displays and photovoltaic systems. Current advances in producing printable ITO inks are reducing the volume of wasted indium during thin film patterning. However, their applicability to flexible electronics is hindered by the need for high temperature processing that results in damage to conventional polymer substrates. Here, we detail the conditions under which laser heating can be used as a replacement for oven and furnace treatments. Measurements of the optical properties of both the printed ITO film and the polymer substrate (polyethylene terephthalate, PET) identify that in the 1.5–2.0 *μ*m wavelength band there is absorption in the ITO film but good transparency in PET. Hence, laser light that is not absorbed in the film does not go on to add a deleterious energy loading to the substrate. Localization of the energy deposition in the film is further enhanced by using ultrashort laser pulses (~1 ps) thus limiting heat flow during the interaction. Under these conditions, laser processing of the printed ITO films results in an improvement of the conductivity without damage to the PET.

## Introduction

Transparent conductive oxide (TCO) films are widely used in a variety of optoelectronic devices, such as solar cells^1,2^, liquid crystal displays (LCD)^3^, touch screens^4,5^, and light-emitting diodes (LED)^6–8^. One of the most popular materials is indium tin oxide (ITO), mainly due to its high transparency in the visible part of the spectrum and relatively low resistivity (in the region 1 − 2 × 10^−4^ ${\rm{\Omega }}\,\cdot \,{\rm{cm}}$)^9^. ITO films can be deposited via chemical vapour deposition^10^, spray pyrolysis^11^, and evaporation^12^ but the most commonly used technique is magnetron sputtering^13,14^. All of these techniques require either a high substrate temperature during deposition (300–500 °C) or a high-temperature (400–700 °C) annealing treatment to meet the transparency and resistivity criteria of most applications. Consequently, the use of polymer substrates is limited in scope as their upper working temperature would be exceeded. Further patterning steps may also compromise the substrate integrity.

Solution-based deposition methods, such as inkjet^15^ and gravure printing^16–19^ are considered to be one of the possible ways to increase material utilization (as lossy post patterning is not required), reduce costs and improve compatibility with the heat-sensitive (e.g. polymer) substrates^20^. Generally, the films produced via these techniques consist of conducting nanoparticles capped with organic stabilizers. Such stabilizers between the particles lead to fewer percolation paths in the films and, in turn, higher resistivity values. This problem is typically addressed by a so-called sintering process, during which conductive neck formation between the particles, accompanied by the removal of stabilizers, occurs. Such thermal sintering has been shown to result in transparency values comparable to that of sputtered films; the resistivity values, however, remained one to two orders of magnitude higher^21–23^. Moreover, such an approach requires high-temperatures (400–600 °C), consequently making it ineffective for heat-sensitive substrates. Apart from conventional heating, there are several low-temperature sintering techniques, including chemical sintering^24^, compression^25^, electrophoretic deposition^26^, hydrothermal crystallization^27^, microwave irradiation^28^, plasma treatment^29^, and photonic sintering^30–32^. The last of these examples^32^ includes laser and intense pulsed light (IPL) sintering^33^, and is of particular interest due to its simplicity^34^, relatively low cost and high throughput^35,36^. The main drawback of the IPL method is that due to the high absorbance of most polymers in the infrared region of spectrum it becomes difficult to implement for flexible substrates^37^. The laser sintering approach heavily relies on the optical properties of nanoparticles in the film; one has to choose the radiation source according to their absorption spectrum. This can be easily achieved for the most commonly used metal nanoparticles (Au^38^, Ag^39^, Cu^31^), whose plasmon resonance peaks are located in the visible part of the spectrum^40^, however sintering of TCO particles becomes a challenging task due to their high transparency in that spectral region. Nevertheless, it has been shown that effective sintering of such nanoparticles is feasible using UV excimer lasers by specifically adding compounds to the ink with high absorption at the incident beam wavelength^41–43^. More recently it has been shown that effective excimer laser annealing of a film consisting solely of ITO nanoparticles is possible^44^. The sheet resistance of the film, however, remained relatively high (500 ${\rm{\Omega }}$/sq) and the exact optical transmission values were not reported. Another possible approach to laser sintering of TCO films is to utilize near-infrared radiation sources (0.75–3 *μ*m). In the spectral region of 1.5–3 *μ*m the absorption of TCO particles is dominated by the presence of free electrons and reaches its maximum values^8^. At the same time, typical polymer substrates remain highly transparent up to about 2 *μ*m^36,37^. Several types of radiation sources are currently available in this spectral region, including erbium doped glass (wavelength of 1.54 *μ*m), Ho:YAG (2.01 *μ*m), and Tm:YAG (2.04 *μ*m) lasers^45^. One can also obtain a laser source with a near IR spectrum by adding a set of filtering optics to one with a wider emission spectrum (e.g. a supercontinuum fibre laser^46^). Despite this, there are very few reports of near-infrared laser sintering of TCO films. One study^47^ investigated the interaction of a CW Erbium fiber laser radiation with ITO films on a high temperature substrate. They showed that such treatment results in a film resistivity decrease to about 1.3 × 10^−3^ ${\rm{\Omega }}\,\cdot \,{\rm{cm}}$^47^. However, as a CW laser was used, it remains unclear whether this method can be applied to polymer substrates with lower working temperatures. This paper investigates the effects of supercontinuum picosecond laser irradiation of ITO films gravure-printed on flexible (PET) foils. The ITO ink consisted of ITO nanoparticles dispersed in a solvent with an addition of a binder^16–18^. The choice of laser allowed selection of the most appropriate wavelength band as informed by the optical measurements. The emission spectrum of the laser was filtered to achieve maximum absorption of the laser light in the ITO particles whilst leaving the substrate undamaged and limiting heat flow due to its picosecond pulse duration. It is shown that such treatment leads to effective sintering of the particles, significantly improving the electronic properties of the film.

## Results and Discussion

### Optical Coupling

**Figure 1:**
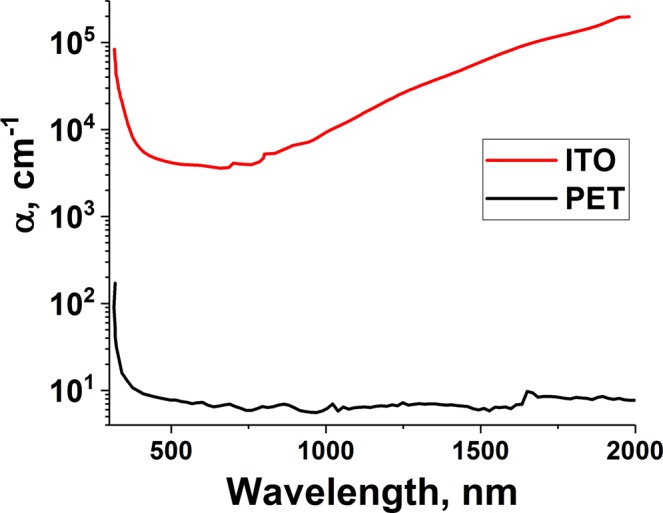
Optical absorption spectra for as-deposited ITO (before laser irradiation) and the PET substrate. The ITO increases in absorption in the near infra red whereas the PET remains transparent.

**Figure 2:**
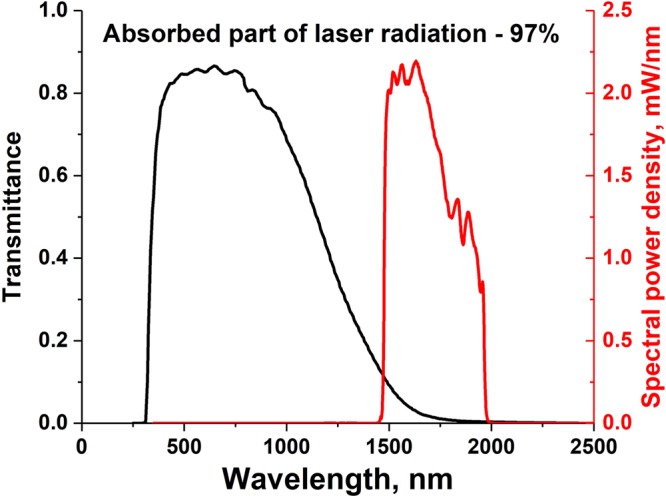
Normalized transmission spectrum of the unirradiated ITO film (black curve) and the emission spectrum of the filtered white light laser radiation (red curve).

The optical absorption coefficients for the ITO film and PET substrate were measured in the wavelength range 250 nm to 2000 nm (Fig. 1). It can be seen that in the visible wavelength region, there is excellent transparency for the PET and, although the unirradiated ITO layer has an absorption coefficient of 3 × 10^4^ cm^−1^, this corresponds to an absorption depth of 2.5 *μ*m which is far in excess of the thickness. For ultraviolet wavelengths, both the film and substrate exhibit strong absorption and any light that does penetrate the ITO will be then absorbed in the PET. This adds an energy loading to the substrate that is to be avoided so as to not compromise its mechanical and optical properties. Whereas, in the near IR, strong absorption is seen in the ink layer whilst the PET remains transparent (Fig. 1). It is for this reason that we have explored laser irradiation using near IR wavelengths and also exploited short pulse durations. The optical energy was supplied by a filtered white light laser (see Experimental section) as shown in Fig. 2. Approximately 97% of the laser energy is absorbed in the film (integrated over the emission spectrum of the laser). Taking 1750 nm as the centre of the filtered laser spectrum, the absorption coefficient of the ITO is 10^5^ cm^−1^ resulting in 5% of the energy reaching the substrate. This energy is then distributed over the entire thickness of PET as the absorption depth (~1 mm) exceeds the substrate thickness of 250 *μ*m.

### Conductivity and Transparency

**Figure 3:**
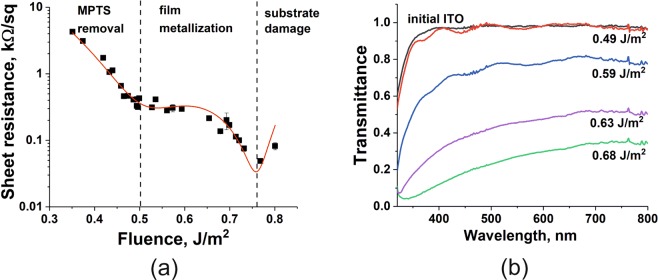
(**a**) Variation of film sheet resistance as a function of incident laser fluence. The irradiation scheme is given in the Experimental section. (**b**) The variation of optical transmission spectrum with incident fluence is shown for conditions that correlate with the plateau region seen in (**a**). As the incident fluence increases, the films become more opaque in the visible region.

**Figure 4:**
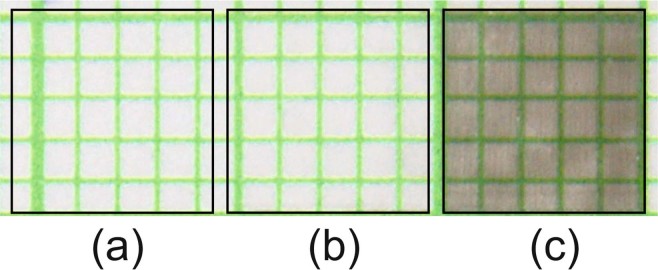
Photographic image of a gravure-printed ITO film irradiated within the areas delineated by the black outlines, at different laser fluence values: (**a**) 0.49 Jm^−2^, (**b**) 0.56 Jm^−2^, (**c**) 0.65 Jm^−2^. Paper with millimetre squares has been used as a background.

**Figure 5:**
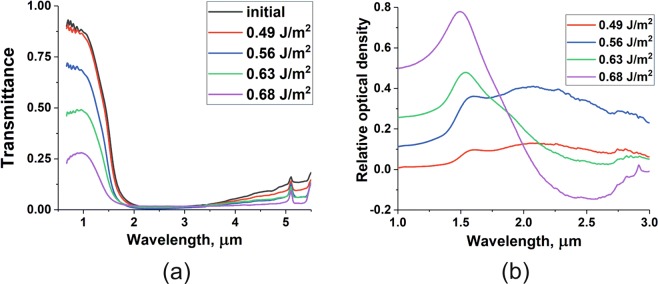
(**a**) Near infra red transmission spectra referenced to the unirradiated film value in the visible. As the incident laser fluence is increased, the transmission at ~1 *μ*m decreases and the cut-off shifts to shorter wavelengths. Expanding this spectral region and plotting relative optical density (**b**), shows the appearance of a resonant peak that is attributed to plasmon resonance effects due to a change in size of the ITO nanoparticles and their local optical environment (i.e. loss of MPTS binder).

The main objective of laser sintering of wet-processed ITO films is to increase their conductivity whilst retaining transparency in the visible spectrum. A series of laser exposures were carried out to investigate the dependence of film sheet resistance on irradiation parameters. The results of such studies are given in Fig. 3(a). It can be seen in Fig. 3(a) that the sheet resistance of the films depends non-monotonously on laser fluence. The line is included to guide the eye and shows that after an initial decrease in resistance of approximately one order of magnitude, the effect is seen to saturate. One can ascribe this phenomenon to removal of the binder (MPTS) causing change in average distance between the ITO nanoparticles, which is known to significantly affect the conductivity of the film^48^. The observed saturation corresponds to the point where no binder is left in the film composition. Increasing the fluence beyond ~0.7 Jm^−2^ causes the resistance to begin to decrease again until damage is observed above 0.75 Jm^−2^ with a concomitant loss of material and conductivity. For most applications, such as electrical contacts for displays or photovoltaic cells, a high level of transparency is required. Figure 3(b) shows that the optical transmission spectra exhibit a very low degree of oscillation in the blue region, indicating good refractive index matching to the substrate. However, the transmission of the film-substrate system becomes compromised as the incident fluence is increased. The irradiation conditions correspond to the plateau region seen in Fig. 3(a). We interpret this behaviour as the film becoming more metallic in character, caused by the laser-induced sintering of ITO nanoparticles. Consequently, its conductivity improves but its opacity increases, as qualitatively shown in Fig. 4. This is often more easily identified in the near infra red as the so-called plasma edge is seen to move to higher frequencies. Therefore, near IR spectra were measured (Fig. 5(a)). It can be seen that there is a subtle shift in the cut-off around 1.5 *μ*m towards shorter wavelengths (higher frequencies). To explore further, this data was re-plotted as optical density relative to the original, unirradiated, film (Fig. 5(b)). Here, a peak in the vicinity of 1.5 *μ*m is seen to grow and shift to shorter wavelength. Whilst the shift was expected due to the increasing conductivity, the appearance of a resonant feature is tentatively attributed to changes in plasmon resonance of the nanoparticle-based ITO film. One should mention that the position of said resonance depends on several factors including the refractive index of surrounding medium, chemical composition, and morphology of the nanoparticles in the film^49,50^. The possibility that the particle morphology has changed (i.e. sintering is occurring) due to the absorption of laser radiation is considered further in the x-ray diffraction section.

### Structure

In order to explore some of the potential mechanisms involved in the laser-induced modification of the as-printed film indicated by the conductivity and optical transmission measurements, further experiments were conducted. These concentrated on the physical structure and composition of the surface layer via x-ray diffraction, Raman spectroscopy and examination by electron microscope.

#### X-Ray Diffraction

**Figure 6:**
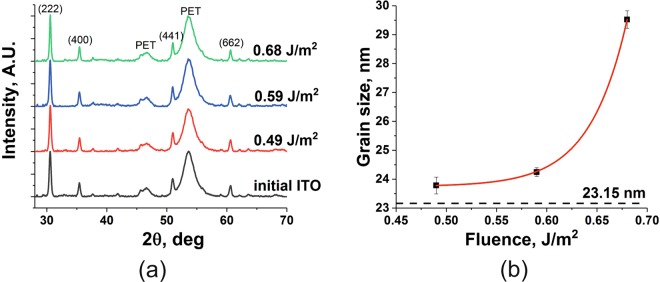
(**a**) X-ray diffraction data for ITO-based ink films on PET irradiated at the fluences indicated. The PET features remain unchanged indicating no structural decomposition of the substrate. The (222) peak associated with the ITO particles is found to narrow as the incident laser fluence is increased. Application of the Scherrer equation to this feature allows calculation of the grain size and is shown in (**b**). The unirradiated value of the ITO particle size based on the XRD data is 23.15 nm and compares well with the average particle size employed in the manufacture of the ink (~25 nm).

The suggestion that nanoparticle sintering is occurring due to the application of laser radiation is supported by the results of x-ray diffraction (XRD) analysis (Fig. 6(a)). As the incident laser fluence is increased, no new diffraction features appear in the pattern. However, careful examination of the strongest peak, the (222) Bragg reflection from ITO, shows that its FWHM decreases by 30% with increasing fluence from 0.59 to 0.69 Jm^−2^. One can then use the Scherrer equation to estimate the approximate grain size of the film components (Fig. 6(b)). It should be noted that the initial grain size calculated this way (23.15 nm) correlates well with the size of the nanoparticles used in the film deposition (~25 nm). Therefore, the increase in grain size is attributed to a corresponding increase in particle size indicating the beginnings of a sintering-based process. It should be noted that the Scherrer equation gives a lower bound for the particle size and so the possibility of the occurrence of larger polycrystalline agglomerates is not excluded by this analysis. The contribution to the XRD data from the semi-crystalline PET substrate is seen to remain unchanged during laser treatment of the overlying ITO film (see features labelled “PET” in Fig. 6(a)). This gives confirmation of by-eye observations that the substrate remains intact during the process.

#### Raman Spectroscopy

**Figure 7:**
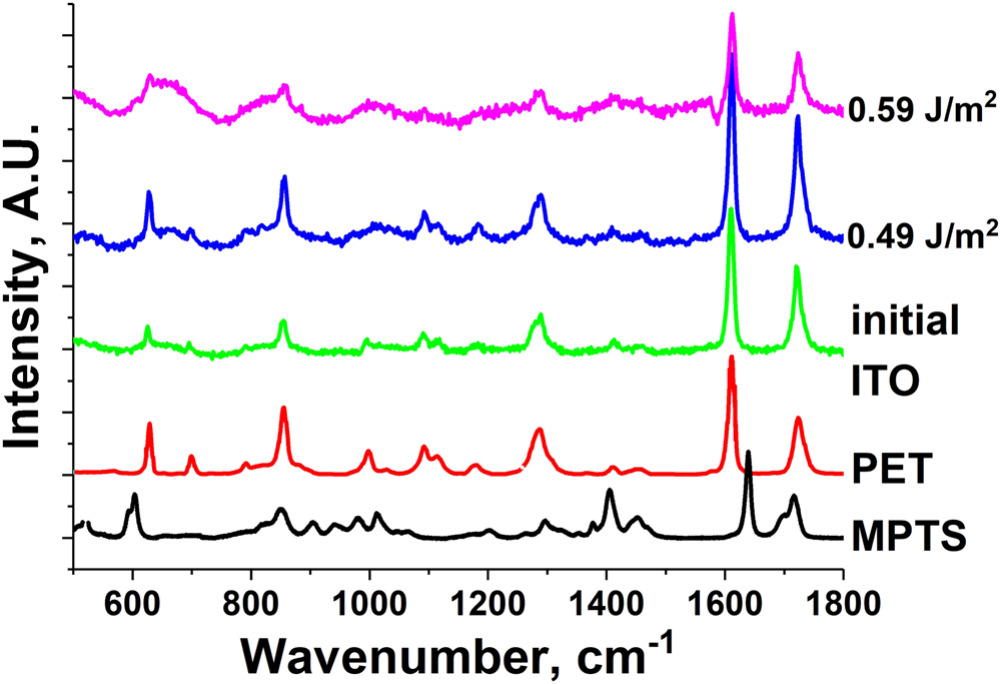
Raman spectra of the ITO films irradiated at different laser fluence values. The PET peaks remain unchanged and there is no appearance of a D-band carbon peak in the spectrum at around 1365 cm^−1^ supporting the conclusion that the substrate is undamaged by the process.

Whilst the XRD data did indicate the presence of semi-crystalline PET, no features associated with the MPTS binder could be observed. As the loss of this dielectric material will have an effect on the electrical conductivity of the film, it was monitored using Raman spectroscopy (Fig. 7). The Raman spectrum of the initial ITO film turned out to largely depend on the properties of the substrate (PET). One can see that no peaks corresponding to MPTS were observed. On the other hand, the presence of the peaks corresponding to the substrate might serve as a good indicator of it remaining undamaged after laser irradiation. It can be seen that in the “working” range of fluences (i.e. where the film transmission remains relatively high) no changes in the substrate Raman spectrum happened. The absence of the D-band carbon peak in the spectrum (at around 1365 cm^−1^) confirms that the laser irradiation does not cause any damage to the PET film via thermal decomposition.

#### Electron Microscopy

**Figure 8:**
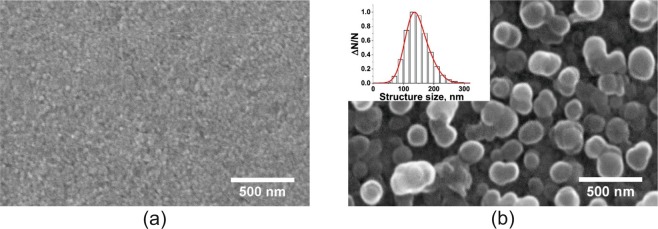
SEM images of (**a**) the unirradiated surface of the ITO film and (**b**) following irradiation at a laser fluence of 0.7 Jm^−2^. The inset in (**b**) shows the transverse size distribution of the surface structures as measured from the SEM micrograph. The smooth unirradiated surface seen in (**a**) is found to become heavily textured in (**b**) as the polymer binder is removed by the laser interaction.

Scanning electron microscope (SEM) analysis of the unirradiated and irradiated ITO ink surface layer was performed. The SEM results (Fig. 8) show that before laser irradiation (Fig. 8(a)), the surface is smooth yet granular with any voids between ITO nanoparticles being filled with MPTS binder. At a fluence of 0.7 Jm^−2^, the polymer binder has largely been removed (Fig. 8(b)). Substantial change in surface morphology is observed and surface structures with transverse size of about 100–200 nm start to appear (see inset of Fig. 8(b)). This is compatible with the XRD grain size analysis which only sets a lower bound on the particle size. It is also possible that the structures observed could be interpreted as ITO nanoparticle agglomerates covered by small amounts of residual organic material. In this regime, metallic behaviour is observed in the form of low visible light transmission but increased electrical conductivity. This hinders the application of this higher fluence for devices that require light input (photovoltaics) or output (displays).

## Conclusions

It is desirable to reduce the usage of indium in transparent conductive oxide films and also to develop processes compatible with low temperature, polymer, substrates. We have shown that gravure printing of inks based on ITO nanoparticles in a polymer binder, followed by laser irradiation, results in electrically conductive films without damage to the PET substrate. In this way, the volume of ITO used has been reduced as compared to large area sputter coating and a polymer substrate has also been able to be utilised. This has been achieved by tailoring the laser source to be strongly absorbed in the ink film whilst any light penetrating the coating is transmitted by the substrate. This reduced energy loading on the PET is also helped by minimising heat conduction during laser irradiation by restricting the laser pulse duration to the picosecond regime. The increase in conductivity due to the action of the laser light can be attributed to the removal of the dielectric MPTS binder in the ink and concomitant particle sintering. This is supported by XRD data that show an increase in grain size and SEM micrographs that indicate larger particles than in the initial sample. In order to maintain a transparency >80%, the present work has found that the sheet resistance does not go below ~300 ${\rm{\Omega }}$sq^−1^. However, if lower transparency can be tolerated, higher laser fluences result in metal-like behaviour before damage of the film occurs and a lower value of ~50 ${\rm{\Omega }}$sq^−1^ can be achieved, albeit at a transmittance of ~20%. The applicability of these films to flexible electronics applications is demonstrated in the Supplementary Information through bending tests.

## Experimental

### Equipment

A Fianium WhiteLase SC400-4 was used as the laser radiation source and has a supercontinuum spectrum ranging from 400 to 2400 nm, pulse duration of 6 ps, maximum power of 4 W and repetition rate of 40 MHz. The emission bandwidth was restricted to a narrower range by means of a dichroic mirror (Thorlabs DMLP1180) and a bandpass filter (Thorlabs FB1750-500) to achieve maximum absorption by the film without damaging the substrate. This filtered radiation was then focused by a 50 mm lens to a 45 *μ*m diameter spot on the target surface. The sample was moved in the stationary beam using Aerotech ALS130 linear translation stages so as to raster a larger area with 25 *μ*m line spacing (55% beam overlap). The resistivity measurements were carried out using a Jandel four-point probe system. Each resistance data point is from a treated area 3 mm × 1 mm. This allowed several resistance measurements (5 per sample) to be taken for one laser irradiated area. Other areas were irradiated in supplementary experiments (e.g. for resistivity as a function of number of pulses, or spot size) and correlation found were the parameters of these distinct studies intersected. The visible range transmission spectra were measured by a Horiba Fluoromax spectrometer. In the near infrared part of the spectrum, a Bruker IFS66 was used. An XRD diffractometer (PANalytical Empyrean) was employed to analyse the crystalline structure changes. Raman spectra of the irradiated samples were acquired by means of a Horiba iHR320 spectrometer with 532 nm excitation wavelength.

### Materials and sample preparation

Initial ITO films were gravure-printed using an ITO ink which consists of crystalline nanoparticles of ITO (In_2_O_3_:Sn, 8 mol% Sn) with an average size of 25 nm, a suitable solvent and a binder. UV-curable printing inks with a 30 wt.% ITO content were prepared by dispersion of the ITO nanoparticles in an alcoholic solvent and addition of 3-methacryloxypropyl-trimethoxysilane (MPTS) as binder which was polymerised after UV irradiation^16–18,51^. For gravure printing a laboratory gravure printing machine (Labratester, Norbert Schläfli Maschinen, Switzerland) was used. The details of the printing process are described in^16–18^. The ITO coatings were dried at 70 °C for 10 min for the transport from INM to Hull before laser treatment. The final film thickness before laser irradiation was 400 nm as determined by stylus profilometry, whilst the PET substrate was 175 μm thick (Melinex® ST504).

## Supplementary information

Supplementary Information
