# Supplementary material for: Laser sintering of gravure printed indium tin oxide films on polyethylene terephthalate for flexible electronics
Source: Sci Rep. 2019 Feb 11;9:1773. doi: 10.1038/s41598-018-38043-y (PMC6370795; doi:10.1038/s41598-018-38043-y)
Supplement: Supplementary file 1 — Supplementary Information [file 41598_2018_38043_MOESM1_ESM.pdf]

Supplementary Information

Laser sintering of gravure printed indium tin oxide films on polyethylene terephthalate for flexible electronics

A A Serkov[1], H V Snelling*[1], S Heusing[2], T Martins Amaral[2]

[1]UNIVERSITY OF HULL, PHYSICS - SCHOOL OF MATHEMATICS AND PHYSICAL SCIENCES, KINGSTON UPON HULL, HU6 7RX, UK

[2]INM – LEIBNIZ INSTITUTE FOR NEW MATERIALS, DEPARTMENT OF OPTICAL MATERIALS, 66123 SAARBRUECKEN, GERMANY

*h.v.snelling@hull.ac.uk

S1. Optical quality of laser-irradiated films

The optical quality of the laser-irradiated films is important for most applications. In the main body of the paper, this has been characterised through transmission spectra and optical photography. Here, we have added supplementary data (figure S1) to illustrate the change in haze as the films are irradiated at increasing laser fluences.

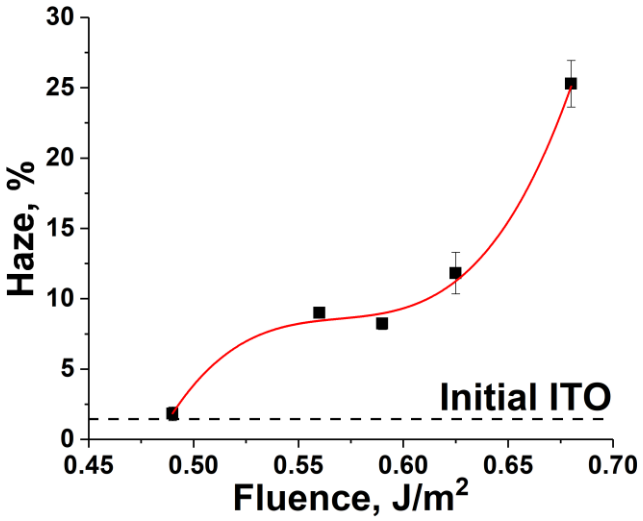

Figure S1. Variation of film haze as a function of incident laser fluence. The dashed line corresponds to the haze value of unirradiated ITO film on PET (approximately 2%).

S2. Surface quality of gravure printed films

The SEM micrographs in the main paper illustrate the change in surface morphology that occurs as the polymer binder is removed by laser irradiation. Here, we show the initial quality of the as-printed films (figure S2) and how the roughness changes with increasing laser fluence (figure S3).

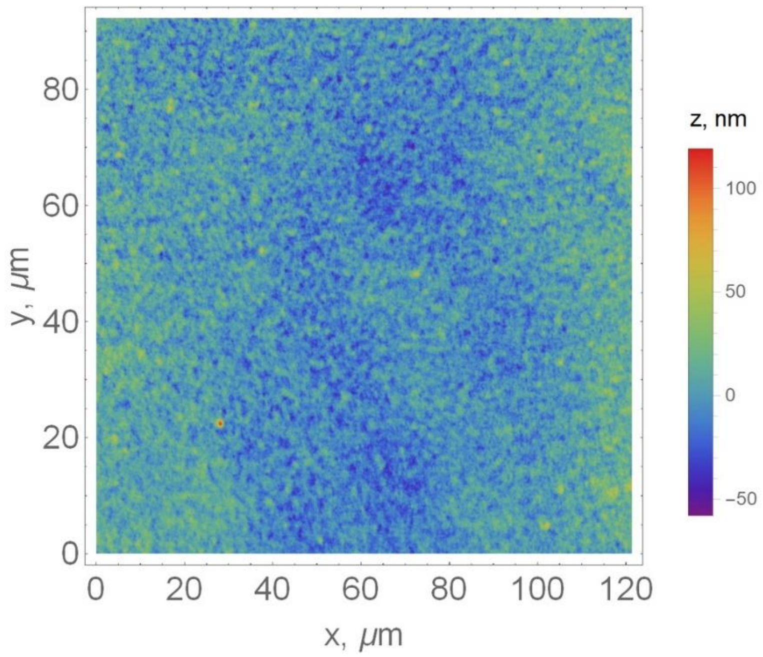

Figure S2. White-light interferometer image of the unirradiated gravure-printed ITO film. The average roughness Ra is of 13.3 nm, the root mean square deviation Rq is of 21.6 nm (values were calculated over 2×2.5 mm area)

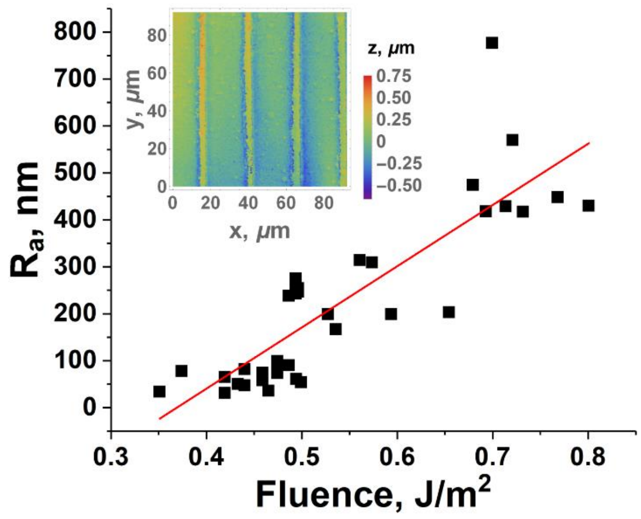

Figure S3. Variation of film average roughness as a function of incident laser fluence. The measurements were carried out for the same samples that were used for the sheet resistance measurements (Fig.3a in the paper). One can see that although the increase in laser fluence corresponds to the growth of surface roughness, it can be mostly ascribed to the traces of beam scanning (see inset, the white-light interferometer image of the ITO film irradiated at 0.73 J/m^2) becoming more pronounced at higher fluence values

S3. Resistivity as a function of bending

One of the applications of transparent conductive films on polymer substrates is in the nascent filed of flexible electronics. We have explored the suitability of the laser irradiated films by performing bending tests on them and characterising any changes in resistivity. Below (figure S4) the data show that for radii of curvature >7.5mm, there is little change in the sheet resistance for a static bend. Repeated bending at this radius (figure S5) can increase the sheet resistance but this effect saturates after approximately 500 cycles and is then stable at ~3 times the original value.

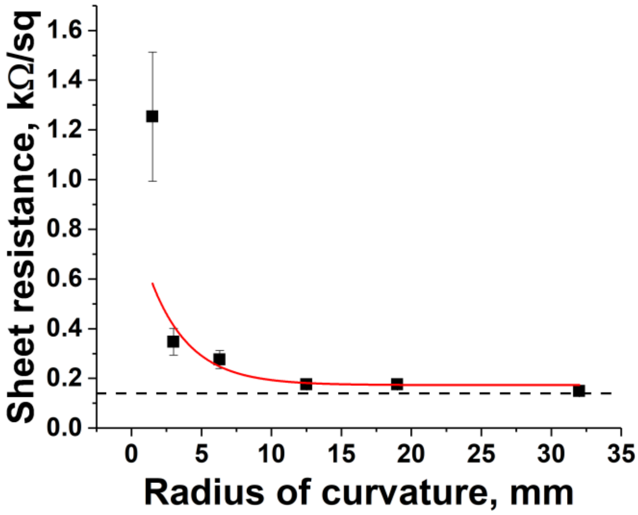

Figure S4. Variation of sheet resistance of an ITO film sample irradiated at 0.7 J/m^2 as a function of the radius of curvature it is bent around. The dashed line denotes the sheet resistance of the sample prior to bending (140 Ω/sq)

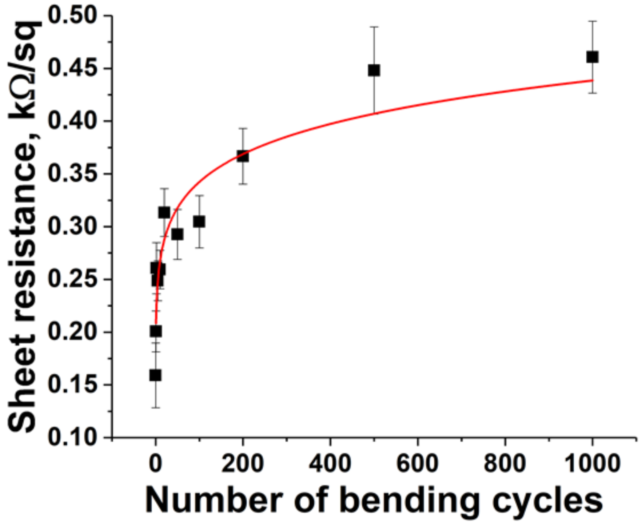

Figure S5. Variation of sheet resistance of an ITO film sample irradiated at 0.7 J/m^2 as a function of the number of bend cycles around a 7.5 mm radius. The sheet resistance value at zero bends corresponds to the sheet resistance of the sample prior to bending (140 Ω/sq)
